# Metabolic Reprogramming by 3-Iodothyronamine (T1AM): A New Perspective to Reverse Obesity through Co-Regulation of Sirtuin 4 and 6 Expression

**DOI:** 10.3390/ijms19051535

**Published:** 2018-05-22

**Authors:** Fariba M. Assadi-Porter, Hannah Reiland, Martina Sabatini, Leonardo Lorenzini, Vittoria Carnicelli, Micheal Rogowski, Ebru S. Selen Alpergin, Marco Tonelli, Sandra Ghelardoni, Alessandro Saba, Riccardo Zucchi, Grazia Chiellini

**Affiliations:** 1 Department of Integrative Biology, University of Wisconsin-Madison, Madison, WI 53706, USA; hreiland5@gmail.com (H.R.); eselinselen@gmail.com (E.S.S.A.); 2 National Magnetic Resonance Facility at Madison, Madison, WI 53706, USA; tonelli@nmrfam.wisc.edu; 3 Department of Surgical Pathology, Medicine, Molecular and Critical Area, University of Pisa, 56126 Pisa, Italy; marti.saba88@gmail.com (M.S.); lorenzini.leo@gmail.com (L.L.); vittoria.carnicelli@discau.unipi.it (V.C.); sandra.ghelardoni@med.unipi.it (S.G.); alessandro.saba@med.unipi.it (A.S.); riccardo.zucchi@med.unipi.it (R.Z.); 4 School of Medicine, Division of Cardiovascular Disease, University of Alabama at Birmingham, Birmingham, AL 35233, USA; rogowskimp@gmail.com; 5 Department of Biological Chemistry, Johns Hopkins University, Baltimore, MD 21205, USA; 6 Department of Biochemistry, University of Wisconsin-Madison, 433 Babcock Drive, Madison, WI 53706-1544, USA

**Keywords:** 3-iodothyronamine, metabolomics, obesity, glucose and lipid metabolism, sirtuins

## Abstract

Obesity is a complex disease associated with environmental and genetic factors. 3-Iodothyronamine (T1AM) has revealed great potential as an effective weight loss drug. We used metabolomics and associated transcriptional gene and protein expression analysis to investigate the tissue specific metabolic reprogramming effects of subchronic T1AM treatment at two pharmacological daily doses (10 and 25 mg/kg) on targeted metabolic pathways. Multi-analytical results indicated that T1AM at 25 mg/kg can act as a novel master regulator of both glucose and lipid metabolism in mice through sirtuin-mediated pathways. In liver, we observed an increased gene and protein expression of *Sirt6* (a master gene regulator of glucose) and *Gck* (glucose kinase) and a decreased expression of *Sirt4* (a negative regulator of fatty acids oxidation (FAO)), whereas in white adipose tissue only *Sirt6* was increased. Metabolomics analysis supported physiological changes at both doses with most increases in FAO, glycolysis indicators and the mitochondrial substrate, at the highest dose of T1AM. Together our results suggest that T1AM acts through sirtuin-mediated pathways to metabolically reprogram fatty acid and glucose metabolism possibly through small molecules signaling. Our novel mechanistic findings indicate that T1AM has a great potential as a drug for the treatment of obesity and possibly diabetes.

## 1. Introduction

**Figure 1:**
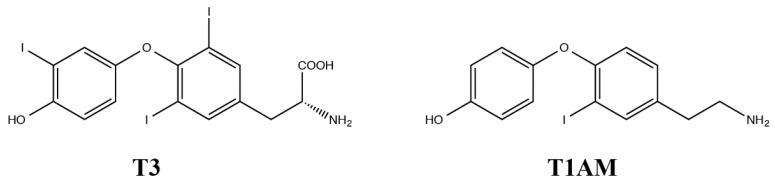
Structures of Thyroid hormone (T3) and 3-iodothyronamine (T1AM).

3-Iodothyronamine (T1AM) (Figure 1) was identified in 2004 as a novel chemical messenger [1], putatively derived from thyroid hormone de–iodination and decarboxylation. T1AM interacts directly with a specific G-protein coupled receptor known as trace amine-associated receptor 1 (TAAR1), which is expressed in brain and in many other tissues [2,3]. Subsequent investigations have confirmed that T1AM can be detected in blood and in most tissues, with concentrations in the nanomolar range [4,5,6]. The number of potential T1AM targets has expanded, in addition to TAAR1, T1AM has been reported to interact with TAAR5 [7], adrenergic receptors [8] and several monoamine transporters [9], suggesting that it should be considered as a multi-targeted ligand. 

The physiological functional responses that were initially observed after administration of exogenous T1AM in the micromolar range included reduced body temperature and cardiac contractility [1,10,11]. However, in subsequent investigations neurologic, endocrine and metabolic responses were elicited at much lower dosages in the submicromolar range that are closer to the physiological range [12,13].

In the hamster and mouse, administration of exogenous T1AM produced persistent weight loss [14,15], raising interest for the potential role of this compound in the treatment of obesity. The use of cavity ring-down spectroscopy (CRDS) and ^13^C-glucose dosing to measure lipid oxidation by real-time breath ^13^CO_2_/^12^CO_2_ (δ^13^C)), showed that an intraperitoneal injection of T1AM (10 mg/kg daily for up to one-week) resulted in a switch from carbohydrate to lipid metabolism lasting for a maximum of four days [15]. The metabolome profiles identified an unusually rapid action of T1AM on both glucose and lipid metabolism associated with weight loss regime, followed by a slower action on lipolysis. However, after day 5 of treatment with T1AM, protein catabolism was stimulated indicating a potential adverse effect of T1AM occurring on a longer time scale.

On the basis of these findings, the purpose of the present study was to provide a comprehensive insight into the metabolic responses to T1AM chronic treatment. We hypothesized that mice given injections of T1AM would experience a switch from carbohydrate to lipid metabolism, leading to observable levels of small molecule lipid intermediates that may impact associated gene expression levels. To test this hypothesis, mice were exposed to two different T1AM dosages (10 and 25 mg/kg) and two different treatment times (4 and 7 days). Since in our previous pilot study we observed that the metabolic effects of T1AM were similar under 4 h fasting and non-fasting conditions [15], in the present study we also introduced a short-term fasting period (4 h), that is adequate to clear immediate diet [16]. Since the effects of T1AM on glucose and lipid metabolism appear to outlast all the other effects, we used nuclear magnetic resonance (NMR) spectroscopy to obtain unbiased metabolomics profiles of tissues and analyzed changes in the expression of genes known to have a significant role in molecular mechanisms relevant to altered glucose and lipid metabolism profiles. In addition, we also employed high performance liquid chromatography coupled with tandem mass spectrometry (LC/MS-MS) to determine the final concentrations of non-metabolized T1AM in different tissues at the end of T1AM treatments to link to its tissue specific physiological effects. 

## 2. Results 

### 2.1. Food Consumption, Activity Levels, Weight Loss and Lipid Profiles

We administered T1AM to spontaneously overweight female Institute for Cancer Research; Caesarean Derived-1 (CD-1^®^) mice. We used two different dosages (10 and 25 mg/kg/day) for 7 days to examine the effects of T1AM on food intake, activity levels, body weight and lipid profiles. No significant differences in food consumption between T1AM-treated and control animals were observed during the study period. Video monitoring did not reveal any noticeable difference in activity level between the three groups of animals. 

Chronic administration of 10 mg/kg/day T1AM showed a 10% body weight loss by day 7 of treatment (Table 1). Body weight loss reached 18% after day 7 of treatment with 25 mg/kg/day T1AM.

During the seven days of treatment control mice only lost 4% of their initial body weight, thus the net body weight loss was 6% after treatment with 10 mg/kg/day T1AM and 14% for 25 mg/kg/day T1AM. Blood samples taken on day 7 revealed that the high dose T1AM treatment produced a significant increase in total plasma triglycerides (67.3 ± 4.23 vs. 49.0 ± 3.46 mg/dL, *p* < 0.05) and a significant decrease in plasma cholesterol (77.7 ± 3.61 vs. 90.8 ± 8.57 mg/dL, *p* < 0.05), without any significant change in glycaemia (Table 1). 

### 2.2. Nuclear Magnetic Resonance (NMR) Metabolome Analysis

#### 2.2.1. Plasma Metabolome

**Figure 2:**
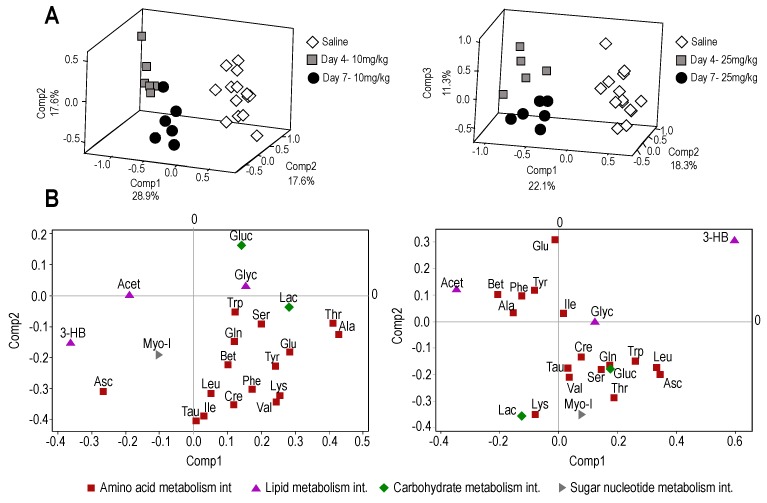
Partial least square discriminant analysis (PLSDA) plots of plasma with and without T1AM treatments show separation of metabolic profiles in obese groups. (**A**,**B**) show PLSDA and score plots for T1AM treatment with 10 mg/kg body weight/day and 25mg/kg body weight/day at two time points (Day 4 and Day 7). Dark gray squares and black circles represent T1AM treatment groups for 4 and 7 days and white diamonds show saline (control) groups. Abbrev: Fatty acid metabolism (black triangles): Glyc, glycerol; Acet, acetate; 3-HB, 3-hydroxy butyrate; antioxidant (Asc, ascorbate) and amino acid metabolism (red rectangular): Tau, taurine; Leu, leucine; Ile, isoleucine; Cre, creatine; Bet, betaine; Gln, glutamine; Trp, tryptophan; Tyr, tyrosine; Ser, serine; Glu, glutamate; Lys, lysine; Val, valine; Thr, threonine; Ala, alanine. Carbohydrate metabolism (green diamonds): Gluc, glucose; Lac, lactate and Myo-I, myoinositol.

Metabolic profiling of plasma using ^1^H-NMR analysis identified 22 metabolites that belong to major metabolic pathways. Partial least squares discriminant analysis (PLSDA) of plasma metabolome profiles showed that treatments with T1AM (10 or 25 mg/kg/day) resulted in clear separations between T1AM treated and control mice at days 4 and 7, indicating marked effects of T1AM treatment on primary metabolism (Figure 2A). Score plots are shown in Figure 2B indicating major contributing metabolites involving carbohydrate, lipid, amino acids, nucleotide and antioxidant metabolism pathways.

**Figure 3:**
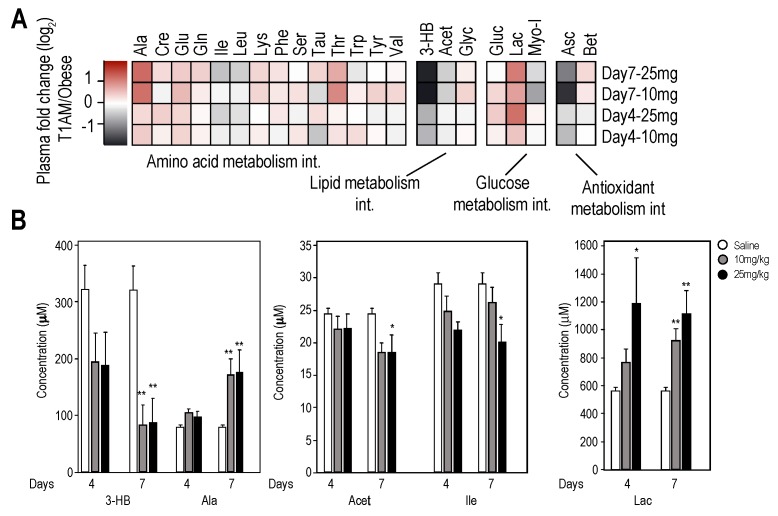
Plasma metabolome profiles. (**A**) Heat map of plasma metabolite fold changes indicates increases or decreases in metabolite concentrations in response to different doses and time periods of T1AM treatments. Differential changes are shown for each identified and quantified metabolite at 10 and 25 mg/kg/day at days 4 and 7. Increased or decreased metabolome levels are color coded for each dose and time point. (**B**) shows bar plots of significantly changed metabolites levels in the T1AM-treated groups (10 mg/kg/day or 25 mg/kg/day) vs. saline group. The *X*-axis shows metabolites ID for Days 4 and 7; and shaded codes are defined as: control mice (white bar), 10 mg/kg/day T1AM-treated mice (gray bar) and 25 mg/kg/day.T1AM-treated mice (black bar). *Y*-axis shows relative concentration levels to formate (internal standard). In all analyses, statistical significance is shown as: * *p* < 0.05, saline vs. treatment, ** *p* < 0.01 saline vs. treatment, respectively.

Heat map representations were used to visualize the magnitude and directionality of change in metabolite levels at two doses and two times points in plasma (Figure 3A) under short-term experimental fasting conditions. 

Collectively, higher dose T1AM treatment appeared to elicit extensive changes in metabolism by increasing or decreasing the level of metabolites involved in major metabolic pathways, including antioxidant metabolic intermediates (Asc and Bet). However, statistically significant changes were only observed for a smaller subset of metabolites belonging to glycolysis (Ala and lactate), lipolysis (acetate and 3-HB) and amino acid metabolism (the branched chain amino acid (Ile) and (Ala)) (Figure 3B). Changes appeared to follow the dose level and the period of treatment with T1AM. Specifically, plasma samples taken at Day 4 and 7 of treatment revealed increasing lactate (Lac) concentrations for both T1AM doses, however, the concentration level of lactate in the low dose T1AM only reached significance on Day 7 of treatment, indicating increased glycolysis. On the other hand, the glucogenic amino acid, Ala showed an increased trend at 10 mg/kg at Day 4 and 7 while reached significance (*p* < 0.05) at 25 mg/kg dose and at Day 7 of treatment as compared to the saline treated group. In addition, the plasma level of the ketone body, 3-hydroxybutyrate (3-HB) and that of an intermediate in lipid metabolism, acetate, dramatically decreased from Day 4 to Day 7 at both T1AM dosages. These results suggest a high demand for these metabolites as carbon sources for energy metabolism under our short-term fasting condition. As expected, 3-HB and acetate levels in the control (saline) groups were not changed from Day 4 to 7, indicating the observed changes are due to T1AM treatment.

#### 2.2.2. Liver and Muscle Metabolome Profiles

**Figure 4:**
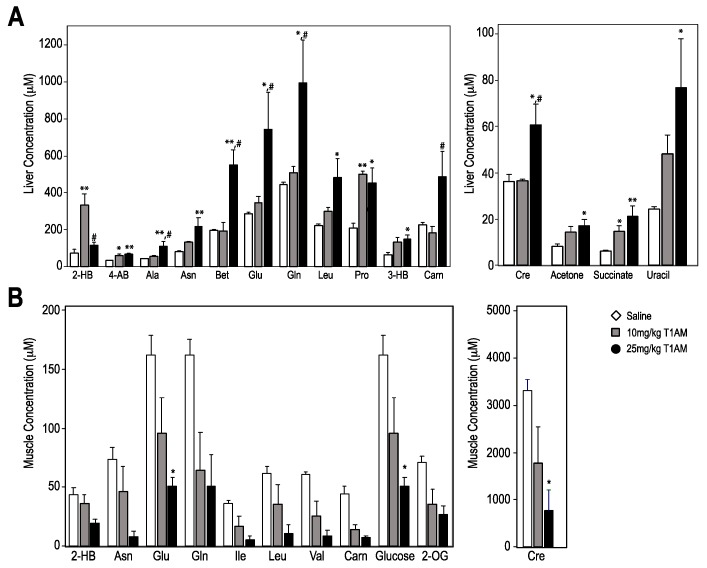
Selected metabolites from liver (**A**) and muscle (**B**) from ^1^H-NMR-based metabolomics profiles at two T1AM doses for seven days treatment. The *X*-axis shows metabolites IDs and the *Y*-axis shows relative concentration levels (M) to formate. Bars are shaded according to each treatment group as: control mice (white), treatment with 10 mg/kg/day (gray) and 25 mg/kg/day (black). The *Y*-axis shows relative concentration levels to formate (internal standard). All data are expressed as the mean ± SEM (standard error of mean). In all analyses, statistical significance is shown as: * *p* < 0.05 vs. saline; ** *p* < 0.01 vs. saline; # *p* < 0.05 10 mg vs. 25 mg.

^1^H-NMR analysis of liver extracts after seven days of T1AM treatment revealed more pronounced dose dependent effects. Indeed, most of the primary hepatic metabolites reached significance at the high T1AM dosage (25 mg/kg). With the exception of 2-hydroxy butyrate (2-HB) all changes in metabolite concentrations showed dose-dependent increases in liver. The observed effects were on lipid catabolism, mitochondrial energy metabolism, amino acid and nucleotide metabolism. In particular, evidence for increased fatty acid oxidation in the liver, the primary site of fatty acid metabolism, comes from increased levels of ketone bodies (3-HB and acetone), carnitine (carn, a fatty acyl carrier that mediates mitochondrial fatty acid oxidation) and succinate (a mitochondrial energy metabolism substrate), as shown in Figure 4A. 

Other primary hepatic metabolites such as amino acids (alanine (Ala), asparagine (Asn), proline (Pro) and leucine (Leu)) and their nitrogen metabolic byproducts (creatine (Cren)) and sugar nucleotide uracil levels were all increased in the T1AM group in a dose-dependent manner. However, changes in liver metabolome profiles for low dose T1AM treatment were less pronounced as compared to the higher T1AM dosage. Only 2-hydroxybutyrate (2-HB), 4-amino-butyrate (4-AB), Pro and succinate reached significance (*p* < 0.05) at 10 mg/kg T1AM dosage. 

^1^H-NMR metabolome analysis of skeletal muscle extracts (Figure 4B) revealed decreasing trends in amino acid and glucose concentrations relative to the saline group. The levels of glucose and amino acids including branched chain amino acids (BCAA, that is, Leu, Ile and Val) were all decreased in a dose dependent manner. In addition, glutamate (Glu), glutamine (Gln), asparagine (Asn) and creatine (Cre) levels were also decreased. Although only Glu and Cre reached statistical significance (*p* < 0.05), these data suggested that T1AM shifts metabolism in muscles towards favoring glucose and amino acids to support its energy demand.

### 2.3. RT-qPCR Results

**Figure 5:**
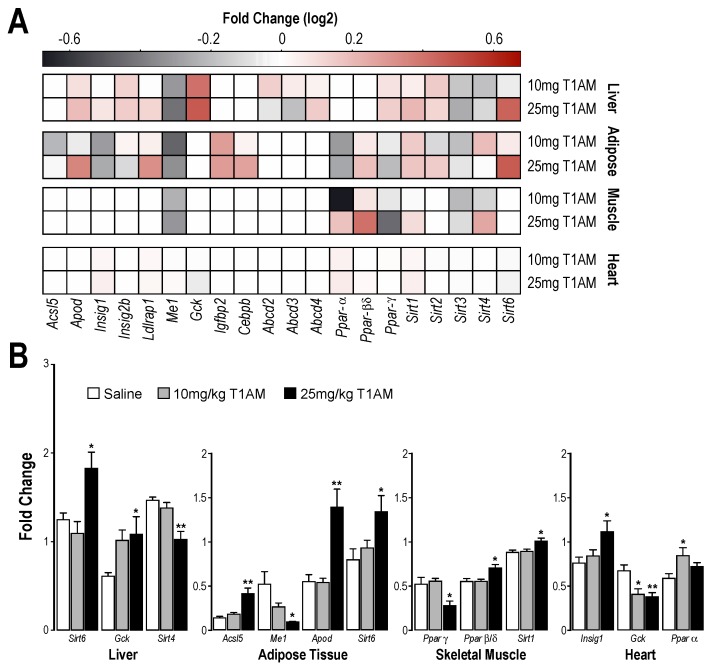
Changes in gene expression evidenced by RT-qPCR in metabolically active tissues. (**A**) Heat map representation of gene expression in liver, adipose tissue, skeletal muscle and heart, respectively. Differential changes in gene expression (Saline treated—T1AM treated) are color coded. Dark and light red shades show > 0; black and gray shades show < 0 in log_2_ base. (**B**) Fold changes in gene expression over the appropriate control are plotted on a log2 scale (*n* = 5). All data are expressed as the mean ± SEM. * *p* < 0.05 vs. saline treated; ** *p* < 0.01 vs. saline treated.

We recently provided the first evidence that T1AM at a subchronic, lower pharmacological dose administration, induced a rapid shift in metabolic pathways from carbohydrate to lipid oxidation [15]. The molecular mechanism by which T1AM favors lipid over glucose catabolism is not known. However, considering the lasting effects of T1AM on fatty acid metabolism [14,15], we speculated that the shift in energy utilization observed in mice after T1AM treatment arises from changes in gene expression. Therefore, targeted gene expression profiles were analyzed in selected metabolically active tissues, including liver, adipose tissue, skeletal muscle and heart, of mice treated with two different T1AM dosages (10 and 25 mg/kg daily up to seven days) as compared with saline treated mice. Our results revealed that at least in tissues principally involved in energy metabolism, such as liver, adipose tissue and skeletal muscle, T1AM administration uniquely impacted the expression of a large set of genes linked to carbohydrate and lipid metabolism (Figure 5A). 

In liver (Figure 5B), T1AM (25 mg/kg/day) significantly up-regulated the expression of genes related to glucose homeostasis and fat metabolism, namely *Sirt6* (sirtuin (silent mating type information regulation 2 homolog) 6) and *Gck* (glucokinase). *Sirt6* regulates glycolysis, triglyceride synthesis and fat metabolism by deacetylating histone H3 lysine 9 in the promoter of many genes involved in these processes [17]. Liver specific deletion of *Sirt6* in mice results in fatty liver formation due to enhanced glycolysis and triglyceride synthesis [18]. *Gck* plays a central role as a glucose sensor in the regulation of glucose homeostasis. In addition, a decreased expression of *Sirt4* (sirtuin (silent mating type information regulation 2 homolog) 4), a gene known to repress fatty acid (FA) oxidation while promoting lipid anabolism [19], was observed in liver after treatment with T1AM (25 mg/kg/day).

In white adipose tissue (Figure 5B), the highest T1AM treatment regime, specifically impacted the expression of genes targeting lipid metabolism and lipoprotein function. *Acsl5* (acyl-CoA synthetase long-chain family member 5), up-regulated by T1AM, is the only ACSL isoform localized on the mitochondrial outer membrane and it is believed to play an important role in the beta-oxidation of fatty acids [20]. *Me1* (malic enzyme 1, NADP^+^-dependent, cytosolic), a lipogenic enzyme that generates NADPH required for fatty acid synthesis, appeared to be down regulated by T1AM. *Apod* (Apolipoprotein D) and *Sirt6* were both up-regulated. APOD is an apolipoprotein structurally related to the lipocalin family proteins that is involved in diverse aspects of metabolism, including lipid transport. Aberrant APOD expression is associated with abnormal lipid metabolism [21]. SIRT6 overexpression has been shown to repress the expression of selected peroxisome proliferator-activated receptor gamma (PPARγ) target genes and key lipid metabolism genes [22] important for triggering lipolysis. A recent report highlighted that SIRT6 deficiency in fat tissue predisposes mice to obesity, insulin resistance and hepatosteatosis [23].

In skeletal muscle tissue (Figure 5B), treatment with T1AM at the highest dose significantly impacted the expression of peroxisome proliferator-activated receptors (*Ppars*), which act as lipid sensors, namely *Pparγ* (related to lipid anabolism, down-regulated) and *Pparβ*/*δ* (related to fatty acid oxidation, up-regulated). Both of these receptors are also linked to SIRT1 protein activity, with *Pparγ* inhibiting *Sirt1* expression, while *Pparβ*/*δ* increasing *Sirt1* mRNA levels, which in turn results in increased lipid oxidation, mitochondrial biogenesis and increased insulin sensitivity [24]. An increased expression level of *Sirt1* was observed consistently in skeletal muscle of the T1AM treated group.

Previous studies showed that T1AM treatment has a protective effect in the heart due to a reduction in oxidative stress [25]. Our results indicate that in the heart, T1AM administration impacted the expression of several genes involved in cardiac energy metabolism. High dose T1AM treatment increased *Insig-1* (insulin-induced gene 1), that is a gene known to have anti-lipogenic action. The glucose level sensor *Gck*, was down regulated at both T1AM dosages, whereas only the lower dosage of T1AM up-regulated *Pparα* gene known to play a major role in the control of cardiac energy metabolism (Figure 5B).

### 2.4. Western Blot of Tissues

**Figure 6:**
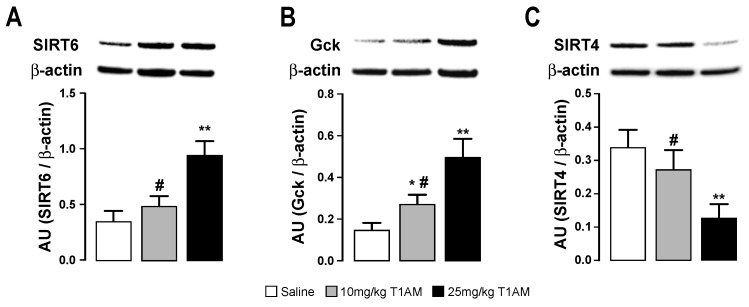
Effects of T1AM treatment on protein expression in liver. Immunodetection for (**A**) Sirtuin (silent mating type information regulation 2 homolog) 6 (SIRT6), (**B**) Glucokinase (GCK) and (**C**) SIRT4 was carried out on protein lysates separated on SDS-PAGE gels. A representative experiment is shown. Results are the mean ± SEM of the densitometry of three different gels. * *p* < 0.05 vs. saline treated; ** *p* < 0.01 vs. saline treated; # *p* < 0.05 T1AM 10 mg/kg vs. T1AM 25 mg/kg.

Our transcriptional analysis results indicated that mice treated with the highest dose of T1AM undergo significant tissue specific changes in gene expression. Protein expression studies by Western blotting confirmed over-expression of sirtuin 6 (SIRT6) and glucokinase (GCK) proteins in liver (Figure 6).

In addition, after treatment with 25 mg/kg/day T1AM, a decreased expression of SIRT4 was also observed (Figure 6). In agreement with the transcriptional findings, the expression of SIRT6 in adipose tissue showed an increasing trend when T1AM was used at the highest dose, however, this trend did not reach statistical significance and it is not reported in Figure 6. 

### 2.5. Tissue T1AM Concentration

The LC/MS-MS assay of T1AM tissue distribution showed that T1AM is distributed in a tissue specific manner (Table 2). 

After 7 days of treatment with 10 mg/kg/day, the remaining concentration of T1AM in adipose tissue, muscle and liver, were 3- to 40-fold higher than controls, respectively. In mice treated for 7 days with 25 mg/kg/day T1AM, a 35- to 100-fold increase was observed in adipose and liver over the control baselines, respectively, whereas in muscle there was only a 20-fold increase as compared to controls. On the other hand, in the heart, the relative amount of T1AM was only about 4-fold higher than control after 7 days of treatment with the highest dose of the drug. 

## 3. Discussion

Recently, we provided the first evidence that sub-chronic treatment with the lowest pharmacological dose of T1AM (10 mg/kg/day) in normal obese mice increased lipolysis associated with significant weight loss but independent of food consumption [15]. In order to get a thorough evaluation of the metabolic response in mice to chronic treatment with T1AM, in the present study we used metabolomics and associated transcriptional gene and protein expression analyses to investigate the tissue specific metabolic reprogramming effects of sub-chronic T1AM treatment (7 days) at two pharmacological doses (10 and 25 mg/kg/day).

Our current observation shows that the net weight loss after treatment with 10 mg/kg/day T1AM for 7 days was 6%, while 7 days of treatment with 25 mg/kg/day T1AM resulted in a 14% net weight loss without affecting any apparent food intake and animal behavior consistent with previous findings [14,15,26]. As expected, the higher dose was more effective at inducing a larger weight loss. Consistent with weight loss and T1AM distribution in tissues, biochemical assays, metabolome profiling and gene and protein expression studies demonstrated significant changes predominantly when the highest T1AM dosage was used and after seven days of treatment.

Multivariate statistical analysis of the ^1^H-NMR based metabolomics data sets reveals marked effects of T1AM treatment on primary metabolism, including amino acid, lipid, carbohydrate and sugar nucleotide metabolism. Increased ketone bodies (3-HB and acetone) in liver were indicative of increased lipid oxidation. Even though no significant differences were observed in plasma glucose levels at both doses, measurement of the plasma metabolome indicated that under the short-term fasting condition, treatment with T1AM induced a high demand for energy metabolism through consumption of glycolytic and lipolytic metabolites. At higher dose (25 mg/kg) of T1AM treatment we observed 3-HB, acetate and Ile were reduced, while lactate was increased when compared to the control group injected with saline. In our previous pilot study [15] the level of 3-HB was increased in plasma under fed condition and injection of ^13^C-glucose at 10 mg/kg T1AM dosage, indicating increased lipid oxidation in plasma despite abundance of glucose for immediate energy metabolism. In both studies, animals did not show increased food consumption, however, under a short-term fasting condition, we postulate that increased level of T1AM treatment elicited an increase in energy demand by other tissues. The observed decrease in 3-HB and acetate plasma levels might indeed reflect increased uptake/consumption by other tissues to meet the higher energy demand. A caveat of the present study is that we cannot completely exclude that the observed effects of T1AM may be partially be induced by the three morning-fasting periods to which the mice were subjected.

Measurement of plasma lipid levels revealed that sub-chronic treatment with T1AM at the highest dose induced a reduction of plasma total cholesterol accompanied by increased triglycerides (TG) levels. The latter increase might be related to an increasing mobilization of triglycerides from the adipose tissue, consistent with increased plasma glycerol levels and in agreement with the metabolic changes related to a higher energy demand after administering T1AM at the highest dose.

Changes in the levels of liver small molecular size metabolic markers (e.g., lactate, acetate, ketone bodies and amino acids), confirm the efficacy of the treatment to induce weight loss. Administration of T1AM at the highest dose (25 mg/kg/day) affected the expression of numerous genes, especially in metabolically active tissues, such as liver, white adipose tissue and skeletal and cardiac muscles. Notably, gene and protein expression findings showed that when T1AM is exogenously administered to mice, it modulates the expression of sirtuins involved in energetic metabolic pathways, namely SIRT6, SIRT4 (in liver and adipose tissue) and *Sirt1* gene (in muscle) through modulation of PPARs (*Pparγ* and *Pparβ*/*δ*). The mammalian sirtuins (SIRT1–7) are a class of NAD^+^-dependent protein deacetylases and/or ADP-ribosyltransferases that regulate a large variety of cellular, physiological and metabolic processes including cell cycle, apoptosis, energy homeostasis, mitochondrial function and longevity [27,28]. Results from recent studies indicate that reduced sirtuin action, as observed with aging and high-fat feeding, is related with Type 2 diabetes. Consequently, sirtuin activators are rapidly emerging as an effective therapeutic strategy against diabetes [29].

Even though the most well-studied sirtuin protein with effects on metabolism is SIRT1, growing evidence indicates that nuclear SIRT6 plays fundamental roles in the maintenance of glucose and lipid homeostasis [30,31]. It negatively regulates glycolysis, triglyceride synthesis and fat metabolism. Accordingly, SIRT6-overexpressing mice are protected from diet-induced obesity and liver-specific deletion of SIRT6 in mice causes fatty liver formation [18,22,23,31].

SIRT4 is a mitochondrial sirtuin that acts as a negative regulator of mitochondrial metabolism. It has been recently observed that SIRT4 inhibition increases mitochondrial function and fatty acid oxidation in liver and muscle cells, suggesting therapeutic benefits for metabolic diseases such as type 2 diabetes [32].

In our study, transcriptional gene and protein expression analysis revealed a significant increase of GCK and SIRT6 expression associated with reduced SIRT4 expression in liver. Even though the molecular target(s) responsible for T1AM functional effects are still largely unknown, these findings support the hypothesis that SIRT6 and SIRT4 might be the mediators of T1AM’s induced shift of metabolism from carbohydrates to lipids through their metabolic intermediates as the principal metabolic activators [15] in this organ. These results suggest that T1AM has great potential to control the balance between glucose and lipid utilization in vivo and open up the way to future pharmacological studies aimed to investigate the hypothesized sirtuin dependency of T1AM metabolic reprogramming in mice. Collectively, our findings are also consistent with the metabolic changes observed by ^1^H-NMR metabolomics of liver and muscle tissues, two high metabolically active organs. To more exhaustively confirm the potential of T1AM as body-weight loss and anti-obesity drug, future studies will be directed to analyze the effects of chronic T1AM administration to mice in combination with chronic aerobic exercise (CAE) or fasting, which are both known to be strong metabolic triggers [33,34,35].

In accordance with previous studies [36], the assay of tissue T1AM concentration (Table 2) suggests that liver and adipose tissue might be regarded as T1AM storage sites and support the elevated metabolic and transcriptional activities observed in both tissues especially when T1AM was administered at the highest dose.

## 4. Materials and Methods

### 4.1. Chemicals

All reagents, unless otherwise other specified, were from Sigma-Aldrich (St. Louis, MO, USA). 3-Iodothyronamine (T1AM) was kindly provided by Thomas Scanlan, Oregon Health & Science University, Portland, OR, USA.

Purified crystalline T1AM was dissolved in 100 μL dimethyl sulfoxide (DMSO) to increase solubility and then diluted ~1:400 with medical grade 0.9% medical grade saline (Hospira Corp., Lake Forest, IL, USA) to minimize toxicity of DMSO. Two final stock concentration levels were made at 25 and 10 mg/kg in 0.9% saline and each solution type was aliquoted and stored at −80 °C until further use.

A vehicle solution was also made for control animals using 100 μL DMSO and 0.9% saline. This solution was injected in control animals (herein referred as “Saline”). 

### 4.2. Animal Study

All animal procedures were approved by the University of Wisconsin, College of Letters and Sciences, Animal Care and Use Committee (Madison, WI protocol # L00408, 12 February 2013), Eighteen out-bred female CD-1 mice obtained from Harlan, (Indianapolis, IN, USA) were used for this study. Female mice are being used in accordance with the previous T_1_AM study [15] to increase the relatability of the results between the two experiments.

Mice were fed an AIN-93G diet (17.7% protein, 60.1% carbohydrate and 7.2 % fat) (Harlan, Indianapolis, IN, USA) and water ad libitum during normal housing conditions and until they reached body weight between 40–45 g for ~4 weeks. Mice were age and weight matched initially between control females and treated with T1AM. These animals were divided into three groups (*n* = 6/group), intraperitoneally injected (i.p.) daily from day 1 to day 7 with: (1) saline solution (control), (2) lower dose T1AM (10 mg/kg/day) and (3) higher dose T1AM (25 mg/kg/day). Mice were injected at 12:00, video monitored from 12:00–14:00 and then were weighed at 16:00. On day 7 of the study, at 16:00 animals were anesthetized using 2.5% isoflurane. Blood was drawn using the retro-orbital venus plexus and heparinized capillary tubes. At this point, animals were sacrificed and their organs collected for NMR-metabolomics, real-time qPCR, Western blot and LC/MS-MS analyses. All tissues were flash frozen in liquid nitrogen and kept at −80 °C until further use.

### 4.3. Plasma NMR Samples Preparation

Blood was drawn on day −3, 4 and 7 of the study through the retro orbital venous plexus using heparinized capillary tubes. Animals were fasted at 08:00 and injected with saline vehicle solution (as described above) on day −3 to collect a baseline plasma sample. On days 4 and 7, food was removed from cages and animals were fasted under the same condition as day −3 at 08:00 and injected with their assigned T1AM treatment at 12:00 and blood was collected at 16:00. Animals had full access to water. Plasma was then separated from blood using centrifugation at 1957× *g* for 10 min and 4 °C. Plasma samples were stored at −80 °C until sample preparation time for NMR. NMR samples from plasma were prepared as previously described [15]. 

### 4.4. Tissue NMR Samples Preparation

Individual 50 mg tissue samples were placed on ice in 10 mM phosphate buffer containing 2 mM sodium orthovanadate, 1 mM NaF, 1 mM phenylmethanesulfonyl fluoride (PMSF) and protease inhibitor cocktail, followed by homogenization by Omni Bead Ruptor Homogenizer (Omni International Inc., Waterbury, CT, USA) for 3 min [37]. Homogenized tissues were transferred to new tubes and were centrifuged for 10 min at 5000× *g*. Supernatant was then transferred to a new tube and ice-cold methanol (2:1, *v*/*v*) was quickly added to aliquots of supernatants, vortexed for 30 s to enhance protein precipitation, followed by cooling to −20 °C for 30 min. After a precipitation period, tubes were vortexed once more for 10 s and centrifuged at 5000× *g* for 10 min. The supernatant was dried in a speed vacuum overnight. The dried supernatant was then reconstituted in the 10 mM phosphate buffer containing 2 mM PMSF, 2 mM ethylenediaminetetraacetic acid (EDTA) and pH adjusted to 7.4 ± 0.05. 

### 4.5. NMR Data Collection and Analysis

All one-dimensional (1D) ^1^H-NMR spectra were collected at 25 °C on a 600 MHz Varian VNMRS spectrometer equipped with a cryogenic probe according to our previously published method [38]. Each 1D spectrum was accumulated for 1028 scans, with an acquisition time of ~2.5 s (24,576 complex points) and a 3 s repetition delay for a total collection time of ~2 h [38]. 1D ^1^H-NMR spectra were referenced to 0.5 mM 4,4-dimethyl-4-silapentane-1-sulfonic acid (DSS). NMR signals arising from small water-soluble metabolites (<1000 Da) were identified and quantified relative to formate (1 mM) as the internal reference by Chenomx software version 6 (http://www.chenomx.com). All metabolite concentrations are reported as values relative to formate.

### 4.6. Tissue Preparation for Gene and Protein Expression Analyses

Liver, subcutaneous adipose tissue, heart and skeletal muscle from the left leg (50 mg/tissue) were rapidly extracted from 18 mice and kept in RNAlater (Qiagen GmbH, Hilden 40724, Germany) for 24 h and stored at −80 °C until use. Tissues were homogenized using a Teflon-glass homogenizer in 1 mL of ice-cold buffer (pH = 7.4) containing 50 mM Tris pH = 7.4, 250 mM NaCl, 5 mM EDTA, 20 mM sodium pyrophosphate, 1% Igepal CA-630, 2 mM sodium orthovanadate, 1 mM NaF, 1 mM PMSF and protease inhibitor cocktail. Homogenates were centrifuged at 3914× *g* for 10 min at 4 °C to pellet cellular debris. The supernatant was collected and frozen to −80 °C. The protein concentration of the supernatant fraction was determined by the Bradford method [39].

### 4.7. Gene Expression Analysis 

Expression of 20 genes (*Acsl5*, *Apod, Insig2a*, *Insig2b*, *Ldlrap1*, *Me1*, *Gck*, *Igfbp2*, *Cebpb*, *Abcd2*, *Abcd3*, *Abcd4*, *Pparα*, *Pparα*/*δ*, *Pparγ*, *Sirt1*, *Sirt2*, *Sirt3, Sirt4*, *Sirt6*) (Table S1) was evaluated in 4 tissues (liver, subcutaneous adipose tissue, skeletal muscle and heart) from the 18 mice by reverse transcription qPCR (RT-PCR). Total RNA was isolated with RNeasy Lipid Tissue Mini kit (Qiagen, GmbH, Hilden, Germany) following the manufacturer’s protocol. 50–100mg of tissue was homogenized in 1mL of QiaZol (Qiagen) with TissueRuptor (Qiagen) for 30–40 s. An on-column DNase treatment with RNase-free DNase Set (Qiagen) was included. RNA concentration and purity (260/280 and 260/230 ratios) were analyzed using a Qubit fluorometer (Life Technologies, Carlsbad, CA, USA) and an ND-1000 Spectrophotometer (NanoDrop Technologies, Wilmington, DE, USA). RNA integrity was checked by agarose gel electrophoresis. 1 µg of total RNA was retrotranscribed in 20 µL (5 min at 25 °C, 20 min at 42 °C, 15 min at 46 °C, 15 min at 50 °C and 5 min at 85 °C) using an iScript cDNA Synthesis Kit (Bio-Rad Laboratories, Hercules, CA, USA).

Relative quantity of gene transcripts was measured by real-time PCR on samples’ cDNA using a SYBRGreen chemistry and an iQ5 instrument (Bio-Rad). Two µL of 25-fold cDNA dilutions and 8 pmol of each oligonucleotide were added to 10 µL SsoAdvanced SYBRGreen Supermix (Bio-Rad) in a 20 µL total volume reaction. The PCR cycle program consisted of an initial 30 s denaturation at 95 °C followed by 40 cycles of 5 s denaturation at 95 °C and 15 s annealing/extension at 60 °C. A final melting protocol with ramping from 65 °C to 95 °C with 0.5 °C increments of 5 s was performed for verification of amplicon specificity and primer dimer formation. 

Primers were designed with Beacon Designer Software v.7.9 (Premier Biosoft International, Palo Alto, CA, USA) with a junction primer strategy. In any case, negative control of retro-transcription was performed to exclude any interference from residual genomic DNA contamination. For quantity data normalization, two to three reference genes were chosen for each tissue type and the values were reported as fold change. Choice was based on testing expression stability of 9 candidate reference genes (*Actb, B2m*, *Gusb*, *Hprt*, *Kdm2b*, *Ppia*, *Psmd4*, *Tbp* and *Rp113*) (Table S2) in tissue specific experiments including all 18 samples. The expression stability of each gene was assessed using geNorm version 3.5 [40]. Efficiency and specificity of primers were tested making standard curves with fivefold serial dilutions of a cDNA obtained from a pool (1 µg) of all mouse liver RNA samples. The first dilution was the two-fold diluted cDNA. All reactions were run in duplicate. Samples were analyzed by the 2^−ΔΔ*C*t^ method as described by Livak and Schmittgen [41]. 

### 4.8. Western Blotting Analysis

Western blotting was performed according to manufacturer’s instructions (Bio-Rad laboratories, Hercules, CA, USA). In brief, 20–40 μg of proteins was subjected to sodium dodecyl sulfate polyacrylamide gel electrophoresis (SDS-PAGE) (Criterion TGX anykD acrylamide separating gel Bio-Rad). The separated proteins were transferred to a polyvinylidene difluoride (PVDF) membrane (Millipore Corporation, Billerica, MA, USA) according to the manufacturer’s instructions. The membranes were dried and then incubated for 1h using primary antibodies for sirtuin proteins (SIRT1, SIRT2, SIRT3, SIRT4, SIRT6), Santa Cruz Biotechnology, Santa Cruz, CA, USA and glucokinase (GCK) Santa Cruz Biotechnology, Santa Cruz, CA, USA) in TBS (20 mM TRIS, pH = 7.6, 137 mM NaCl), 0.04% Tween-20 and 5% low fat milk and then incubated for 30 min with secondary antibodies conjugated with horseradish peroxidase (anti-rabbit, Santa Cruz Biotechnology, Santa Cruz, CA, USA). After washing with TBS, immunoblots were visualized by means of a chemiluminescence reaction (Millipore) by Image Lab^TM^ Software (Bio-Rad) under a luminescent image analyzer (Chemidoc XSR + Bio-Rad, Philadelphia, PA 19103, USA). Only bands below the saturation limit were analyzed and shown. β-actin (Sigma-Aldrich, S.r.l., Milan, Italy) was used as loading control.

### 4.9. LC/MS-MS Assay

T1AM was assayed in liver, abdominal adipose tissue, skeletal muscle and heart by high performance liquid chromatography (HPLC; LC) coupled with tandem mass spectrometry (MS-MS). Liver, heart and skeletal muscle samples (10–50 mg) were homogenized on ice in 1.5 ml of phosphate buffer (154 mM NaCl, 6.7 mM NaH_2_PO_4_, pH 7.4) by 15 + 15 passes in a Potter-Elvejheim homogenizer. The homogenate was centrifuged for 10 min at 18620× *g*, the pellet was discarded and the supernatant was placed in a 15 mL centrifuge tube. After vortexing, 60 mg of NaCl was added; the mixture was equilibrated at room temperature for one hour and then de-proteinized with 2 mL acetone in an ice bath for 30 min. After centrifugation for 15 min the supernatant was evaporated to 1 mL using a Concentrator Plus (Eppendorf, Hamburg, Germany) kept at 30 °C. Subsequent steps included solid phase extraction, HPLC separation and MS-MS assay, which were performed as previously described [4]. Adipose tissue samples (10–50 mg) were extracted for 30 min in 1 mL of acetonitrile and 0.1 M HCl (85:15, *v*/*v*), in an ultrasound bath (LBS1 3Lt, Falc Instruments, Treviglio, Italy). The material was diluted to 2 mL with acetonitrile and homogenized by 12 + 12 passes in a Potter-Elvejheim homogenizer. The homogenate was further sonicated for 10 min with 1510 Branson and a frequency of 40 kHz, then vortexed for 1 min and centrifuged at 720× *g* for 15 min. The supernatant was subjected for three times to liquid/liquid extraction with 1 mL hexane: the upper phase (hexane) was discarded and the lower phase (acetonitrile) was eventually dried under a gentle stream of nitrogen. The dried samples were reconstituted with 0.1 M HCl/methanol (50:50, *v*/*v*) and subjected to HPLC separation and MS-MS assay, as previously described [4]. 

### 4.10. Biochemical Assays

Plasma samples were prepared at the time of sacrifice as previously described [37]. Plasma total cholesterol (Wako Diagnostics, Richmond, VA, USA) and total triglycerides (Sigma Aldrich, St. Louis, MO, USA) were measured using specific colorimetric kits (Table 1).

### 4.11. Statistical Analysis

Data analysis was performed by Graph-Pad Prism 6.0 statistical program (GraphPad Software Inc., San Diego, CA, USA). All data were reported as the mean ± SEM (standard error of mean). The threshold of statistical significance was set at *p* < 0.05 unless otherwise specified. For biochemical assays, gene expression, Western blot and LC/MS-MS, statistical significance was assessed by one-way analysis of variance (ANOVA), followed by Dunnett’s post hoc test, or Tukey’s multiple comparison post hoc test. 

#### Metabolomics

Prior to statistical analyses, all data were examined for assumptions of normality and homogeneity of variance using metaboanalyst procedures (www.metaboanalyst.ca). To evaluate major metabolic changes contributing to separation of metabolome profiles in plasma and tissues under different treatments conditions (T1AM dosages and saline) and their time-dependent changes, we first used a partial least square discriminant analysis (PLSDA) [42,43,44] (www.metaboanalyst.ca). Heat maps were generated based on the fold changes between metabolome profiles of T1AM-treatment and control groups using R statistical software program (http://www.R-project.org). Fold change is defined by ratio of a given metabolite concentration in the control group, [metabolite]_saline_, and the corresponding metabolite concentration in the T1AM treatment group, [metabolite]_T1AM-treatment_. Color codes were used to indicate increase or decrease of each metabolite concentration. Finally, to determine the statistical significance in plasma metabolite profiles, collected at two time points (i.e., Day4 and Day7), two-way analysis of variance (ANOVA) was used followed by Tukey Honest Significant Differences (HSD) test. For tissue (liver and muscle) metabolomics data set, ANOVA followed by Tukey’s multiple comparison post hoc test were performed. In all analyses * indicated *p* < 0.05, control versus treatment; ** indicated *p* < 0.01, control versus treatment; # indicated *p* < 0.05, 10 mg versus 25 mg. Data were presented as standard error measurement (± SEM) for relative concentrations of metabolites to the internal standard (formate).

## 5. Conclusions

Our multidisciplinary approaches provide consistent multiple perspectives on evidence that treatment with exogenous T1AM affects lipid metabolism in a dose-dependent and tissue-specific manner. Taken together, the identification of SIRT6, as well as SIRT4, as potential targets for T1AM-mediated metabolic regulation, coupled with a better knowledge of T1AM tissue distribution and accumulation, has the potential to open broad new avenues in the treatment of a wide variety of diet- and age-related diseases. 

## Figures and Tables

**Table 1:** Effect of T1AM treatment on weight, serum cholesterol, triglyceride and glucose in spontaneously overweight female CD-1 mice.

Assay	Saline	10 mg/kg/day	25 mg/kg/day
Weight loss (g)	−1.6 ± 2.2	−3.8 ± 0.7 ^b^	−8.1 ± 3.2 ^a^
Serum Cholesterol (mg/dL)	90.8 ± 8.57	86.7 ± 7.84	77.7 ± 3.61 ^a^
Serum TG (mg/dL)	49.0 ± 3.46	52.11 ± 10.61	67.3 ± 4.23 ^a^
Serum glucose (mg/dL)	151 ± 21.60	165 ± 35.74	161.52 ± 24.02

Data are shown as mean ± SEM. Significance assigned by ^a^ *p* < 0.05 and ^b^ *p* < 0.07 versus control.

**Table 2:** T1AM distribution in tissues by liquid chromatography mass spectrometry (LC/MS-MS).

Tissue	Saline	Low T1AM (10 mg/kg/day)	High T1AM (25 mg/kg/day)
Liver ^+^	7.68 ± 0.85	318.3 ± 35.09 ^a^	767.0 ± 165.40 ^b^
White Adipose ^+^	0.493 ± 0.17	1.71 ± 0.30 ^b^	16.96 ± 3.96 ^b^
Muscle ^+^	19.84 ± 3.57	56.52 ± 12.64	412.86 ± 109.14 ^b^
Heart ^+^	18.15 ± 4.38	37.62 ± 0.76	68.19 ± 14.10 ^b^

^+^ Concentration of T1AM (pmol/g) determined in tissues of saline and T1AM treated mice. ^a^ *p* < 0.01 vs. control; ^b^ *p* < 0.05 vs. control.

## Supplementary Materials

Supplementary materials can be found at http://www.mdpi.com/1422-0067/19/5/1535/s1.
