# Supplementary material for: Metabolic Reprogramming by 3-Iodothyronamine (T1AM): A New Perspective to Reverse Obesity through Co-Regulation of Sirtuin 4 and 6 Expression
Source: Int J Mol Sci. 2018 May 22;19(5):1535. doi: 10.3390/ijms19051535 (PMC5983833; doi:10.3390/ijms19051535)
Supplement: Supplementary file 1 [file ijms-19-01535-s001.zip › ijms-301382-supplementary.pdf]

Supplementary Material: Metabolic Reprogramming by 3-Iodothyronamine (T1AM): A New Perspective to Reverse Obesity through Co-regulation of Sirtuin 4 and 6 Expression

Fariba M. Assadi-Porter, Hannah Reiland, Martina Sabatini, Leonardo Lorenzini, Vittoria Carnicelli, Micheal Rogowski, Ebru S. Selen Alpergin, Marco Tonelli, Sandra Ghelardoni, Alessandro Saba, Riccardo Zucchi and Grazia Chiellini

Gene expression study

Expression of genes (Table S1) was evaluated in 4 tissues (liver, subcutaneous adipose tissue, skeletal muscle, and heart) from the 18 mice by reverse transcription qPCR. Two to three reference genes were chosen for each tissue type as reference genes [1]. Each choice was based on testing expression stability of 9 candidate reference genes (Table S2) in tissue specific experiments including all 18 samples. The expression stability of each gene was assessed using geNorm version 3.5 [2].

Table S1. primer sequences of target genes.

Gene Symbol	RefSeq mRNA	Forward Primer	Reverse Primer
acsl5	NM_027976	5′-ATCCCTGTGGTCATCTGT-3′	5′-GCCTATATTCTCCGCATCAT-3′
apoD	NM_007470	5′-TGGAGAACGGAAACATCG-3′	5′-GTGGCATCAACGGGAAGA-3′
insig2a	NM_178082	5′-CAATGAATGTACTGAAGGATTT-3′	5′-GAAGTGAAGCAGACCAAT-3′
insig2b	NM_133748	5′-CCGGGCAGAGCTCAGGAT-3′	5′-GAAGCAGACCAATGTTTCAATGG-3′
ldlrap1	NM_145554	5′-CCAAGGAAGAGAAAGAGA-3′	5′-CGCTGACTGTAGATAACG-3′
me1	NM_001198933	5′-TAACGATGATATTCAAGGAACA-3′	5′-TAACAACCAAGTGAGCAAT-3′
Gk	NM_010292	5′-TGCGGAGATGCTCTTTGA-3′	5′-TTGTCTATGTCTTCGTGCCTTA-3′
igfbp2	NM_008342	5′-CCCTGGAACATCTCTACT-3′	5′-GTATTGGGGTTCACACAC-3′
Cebp	NM_009883	5′-CGGGTTTCGGGACTTGAT-3′	5′-CCGCAGGAACATCTTTAAG-3′
abcd2	NM_011994	5′-TCGTGTATGCCACTGCTAA-3′	5′-TCATTTCTACCTTATGTCCTCTGT-3′
abcd3	NM_008991	5′-AGAATGGCGATGGCAAGAC-3′	5′-GTGAAACGGTAAAGAGGGTGAT-3′
abcd4	NM_008992	5′-GAGGATTGTGAGGTTCTTG-3′	5′-CAGGACATCATACCAGTTC-3′
Ppara	NM_001113418	5′-CCACGAAGCCTACCTGAAG-3′	5′-GCCATACACAAGGTCTCCAT-3′
Pparbd	NM_011145	5′-TGCTGTCAAGTTCAATGC-3′	5′-CTGGATGGCTTCTACCTG-3′
Pparg	NM_001127330	5′-CTCACAATGCCATCAGGTT-3′	5′-TCGCAGATCAGCAGACTC-3′
sirt1	NM_001159589	5′-CAGAACCACCAAAGCGGAAA-3′	5′-GCAAGGCGAGCATAGATACC-3′
sirt2	NM_001122766	5′-CAGTTCAAGCCAACCATCT-3′	5′-CTCGTTCCAGCGTGTCTA-3′
sirt3	NM_001177804	5′-CCAATGTCACTCACTACTTCC-3′	5′-TCCCAGATGCTCTCTCAAG-3′
sirt4	NM_133760	5′-GTGTGAAAGAGGCGGACT-3′	5′-GTGAGGATGAACCTGTAACCA-3′
sirt6	NM_001163430	5′-CCAAGTGTAAGACGCAGTA-3′	5′-TCCTCCCAGTCCAGAATG-3′

Table S2. primer sequences of reference genes.

Gene Symbol	RefSeq RNA	Forward Primer	Reverse Primer
actb	NM_007393	5′-GCCTTCCTTCTTGGGTAT-3′	5′-GGTCTTTACGGATGTCAAC-3′
b2m	NM_009735	5′-TGGTCTTTCTGGTGCTTGTC-3′	5′-AGTTCAGTATGTTCGGCTTCC-3′
gusb	NM_010368	5′-CGGTTGTGATGTGGTCTGT-3′	5′-GGCTTTGGTGTGGGTGAT-3′
hprt	NM_013556	5′-TGATTATGGACAGGACTGAAAGA-3′	5′-CAGCAGGTCAGCAAAGAAC-3′
kdm2b	NM_013910	5′-TGGAAGAGGAAGAAGGCAAGTT-3′	5′-GTTGACCACACCCTCCGATT-3′
ppia	NM_008907	5′-GACTGAATGGCTGGATGG-3′	5′-ATCTTCTTGCTGGTCTTGC-3′
psmd4	NM_008951	5′-GAAGGAGGCAAGATGGTGTT-3′	5′-CGGGTGGGAAGGAAGTCT-3′
tbp	NM_013684	5′-GCCTTCCACCTTATGCTC-3′	5′-AGTAAGTCCTGTGCCGTAA-3′
rpl13	NM_016738	5′-GTGAGGTGCCCTACAGTGAGATAC-3′	5′-GATGGTGCGAGCCACTTTCTTG-3′

Reference

1. Kouadjo, K.E.; Nishida, Y.; Cadrin-Girard, J.F.; Yoshioka, M.; St-Amand, J. Housekeeping and tissue-specific genes in mouse tissues. *BMC Genom.* **2007**, *8*, 127.
2. Vandesompele, J.; de Preter, K.; Pattyn, F.; Poppe, B.; van Roy, N.; de Paepe, A.; Speleman, F. Accurate normalization of real-time quantitative RT-PCR data by geometric averaging of multiple internal control genes. *Genome Biol.* **2002**, *3*, research0034.1.
